# Analysis of the utilization, processes, and outcomes of inpatient mental healthcare during the first three waves of the COVID-19 pandemic in the federal state of North Rhine-Westphalia, Germany

**DOI:** 10.3389/fpsyt.2022.957951

**Published:** 2022-12-22

**Authors:** Jürgen Zielasek, Isabell Lehmann, Jürgen Vrinssen, Euphrosyne Gouzoulis-Mayfrank

**Affiliations:** ^1^LVR-Institute for Healthcare Research, Cologne, Germany; ^2^Medical Faculty, Heinrich Heine University Düsseldorf, Düsseldorf, Germany; ^3^LVR Clinic Cologne, Cologne, Germany

**Keywords:** COVID-19, pandemic, psychiatric services utilization, mental healthcare, inpatient

## Abstract

**Background:**

During the first phase of the Coronavirus-19 disorder (COVID-19) pandemic in the spring of 2020, utilization of inpatient mental healthcare was significantly reduced. We now report on a long-term observational study of inpatient mental healthcare in a large psychiatric hospital association in North Rhine-Westphalia, Germany, covering the second and third pandemic waves of autumn and winter 2020 followed up until June 2021.

**Objectives:**

Analysis of the changes of inpatient and day patient mental healthcare utilization in an association of psychiatric hospitals during the COVID-19 pandemic from January 2020 until June 2021.

**Materials and methods:**

We used the statistics database of the association of the nine psychiatric hospitals of the Rhineland Regional Council (Landschaftsverband Rheinland, LVR). We compared the case numbers of the pandemic period with previous years and analyzed changes in the diagnostic spectrum, rates of coercion and therapeutic outcomes. We also analyzed age, gender, diagnoses and coercive measures of patients tested positive for COVID-19 during inpatient psychiatric healthcare.

**Results:**

Case rates were reduced during and after the COVID-19 pandemic episodes of 2020 and the following months of spring and summer 2021. Changes varied between diagnostic groups, and there were even increases of case numbers for acute psychotic disorders. Coercive measures increased during the pandemic, but therapeutic outcomes were maintained at the pre-pandemic level. Women and patients of higher ages were overrepresented among psychiatric inpatients with COVID-19.

**Conclusion:**

The COVID-19 pandemic led to over during reductions of inpatient psychiatric hospital admissions and changes of the diagnostic spectrum accompanied by increased rates of coercive measures. These effects may reflect an overall increased severity of mental disorders during the COVID-19 pandemic, deferrals of inpatient admissions or a lack of outpatient mental healthcare services utilization. To differentiate and quantitate these potential factors, further studies in the general population and in the different mental healthcare sectors are needed. In order to reduce the number of COVID-19 cases in psychiatric hospitals, vaccination of people of higher ages and with dementias seem to be the most needed strategy.

## Introduction

The coronavirus-19 (COVID-19) pandemic had an overall negative impact on the mental health of the general population (1, 2). Due to its medical and social consequences, the COVID pandemic has been traumatizing the general population for long time periods and increased the need of mental healthcare (3, 4). However, the individual mental health responses were heterogenous with a subgroup of approximately one third of the population showing increasing levels of anxiety and/or depression during the first year of the pandemic (5). Ahrens and coworkers (6) described marked interindividual differences in perceived stress between subgroups. In vulnerable groups, psychological state deteriorated over time, putting them at risk for mental disorder development. Consequently, health services should especially identify and allocate resources to vulnerable individuals (6).

Analyzing the time pattern of service utilization during a pandemic is of central importance to prepare services for future pandemics. For example, in a recent UK survey, Bu and coworkers (7) found that mostly friends and families were approached when people in lockdown faced mental health problems. Until now, there have been few reports about the utilization of inpatient mental healthcare during the pandemic. In an earlier study, we showed a reduction of overall inpatient psychiatric admission rates accompanied by a shift toward more acute cases during the initial pandemic phase of early 2020 in Germany (8). Some mental health services were reorganized to include telepsychiatry (9–11). An Italian study indicated an increased demand for urgent psychiatric consultations predominantly for vulnerable groups like people living in psychiatric facilities (12). Following the first pandemic wave in early 2020, two more waves developed in the course of 2020 and 2021. Emergency psychiatric admissions increased even after lockdown measures were curtailed, indicating over during effects of the pandemic and lockdown measures on mental healthcare services (13, 14). Therefore, we were interested in performing a follow-up study of psychiatric hospital admission rates spanning the subsequent pandemic phases in 2020 and 2021. Besides admission rates, we analyzed the long-term numbers and diagnoses of inpatients with mental disorders and COVID-19 comorbidity. These analyses should help to assess the impact of the COVID-19 pandemic on psychiatric inpatient utilization and to clinically characterize psychiatric inpatients with mental disorders who develop COVID-19 infections. Such analyses are expected to be helpful to prepare mental healthcare systems for future virus pandemics.

We studied admission rates and admission diagnoses of adult psychiatry departments of nine psychiatric hospitals of the hospital association of the Rhineland Regional Council [Landschaftsverband Rheinland (LVR)], which have approximately 2,835 beds and 750 day patient places. These hospitals provide mental healthcare services to approximately half of the population of the Rhineland (4.4 million inhabitants) and are representative of mental healthcare services in Germany as shown by their typical day mix indices (8).

## Materials and methods

The observation period for this study was 1 January 2018 until 30 June 2021. This observation period covered the first minor phase of the COVID-19 pandemic in Germany in spring 2020, which was followed by a period of low COVID case numbers in summer 2020 and then by a two more pronounced waves of COVID 19 cases from fall 2020 until early spring 2021. Numbers of COVID-19 cases then again decreased in summer of 2021. We therefore compared the time period 1 January 2018 until 30 June 2019, with the time period 1 January 2020 until 30 June 2021.

Clinical routine data were obtained from the LVR statistics database. These data are documented for clinical routine purposes of quality insurance and remuneration. They included age and sex, diagnoses, length of stay, frequency of coercive measures (involuntary admission, restraint and seclusion) and COVID-19 test results. The clinical routine data of the nine psychiatric hospitals are transferred on a daily basis to an anonymized statistics database, which serves quality assurance purposes. We analyzed the anonymized aggregated data of the departments of general psychiatry, addiction medicine, geriatric psychiatry and psychosomatic medicine by means of time series analysis of the monthly inpatient and day patient admission rates, frequencies of diagnoses of mental disorders, gender and age of patients. We also studied the number, age and gender of inpatients with COVID-19 as identified by the corresponding ICD-10-codes in the statistics database. Additional analyses addressed measures of coercion as identified by the rates of involuntary hospitalizations under the auspices of the North Rhine-Westphalia Mental Health Act, and the case-based rates of coercive measures (seclusion and restraint). For statistical analyses, we calculated odds ratios to assess the risk of COVID-19 infection in different groups of patients. We used Chi-square tests to compare rates of categorical variables between groups and used Student’s *t*-test to compare the age distributions of people with and without COVID-19. Statistical analyses were performed using standard statistical software (SPSS 26, Konstanz Information Miner and GraphPad Version 9.3.1). We performed this retrospective epidemiological study following the principles of the Helsinki Declaration.

## Results

**FIGURE 1:**
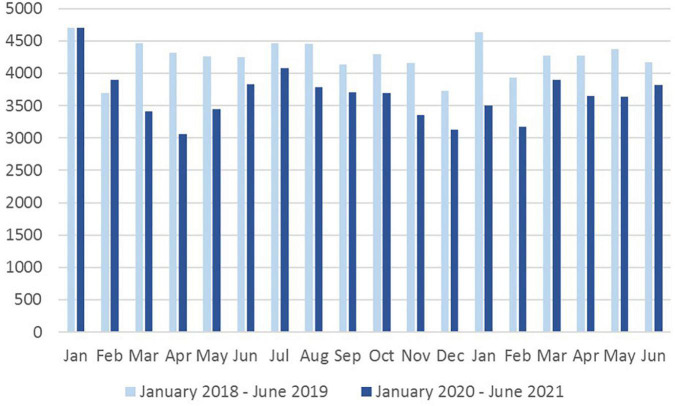
Monthly admission rates. Shown are the monthly admission rates in the adult psychiatric departments of nine psychiatric hospitals of the Rhineland region in Germany in the time periods 1 January 2018 until 30 June 2019 (light blue bars) and 1 January 2020 until 30 June 2021 (dark blue bars).

Comparing the monthly inpatient and day patient admission rates for two 18-months observation periods, there was a decline of 12.5% in inpatients and 25.9% in outpatients (Figure 1). Numbers decreased already in March 2020, when the first COVID 19 pandemic wave occurred in Germany, and similarly so in summer 2020 coincidentally with the second wave. Admission rates never fully recovered to the pre-pandemic values throughout the observation period. The decline of case numbers affected nearly all diagnostic groups, but was different between diagnostic groups. Table 1 shows the changes for the 10 most frequent diagnoses of 2018/2019. The decline was most pronounced among those with affective and substance related disorders. Delirium superimposed on dementia (ICD-10 diagnostic group F05.1) increased slightly and a relatively large increase of 18% was observed for patients with acute polymorphic psychotic disorders (ICD-10 diagnostic group F23.1 from 539 to 636 cases).

**TABLE 1:** Change in the frequency of the 10 most frequent admission diagnoses of the 2018/2019 period.

	2018/2019	2020/2021	Difference	Change
F10.2	13,308	10,740	−2,568	−19%
F20.0	9,803	8,975	−828	−8%
F33.2	9,316	8,170	−1,146	−12%
F32.2	8,025	6,182	−1,843	−23%
F11.2	4,075	3,154	−921	−23%
F05.1	2,298	2,397	+99	+4%
F60.31	2,231	2,126	−105	−5%
F33.1	1,941	1,808	−133	−7%
F10.3	1,757	1,062	−695	−10%
F43.2	1,666	1,355	−311	−19%

We assessed the main discharge diagnoses in nine psychiatric hospitals of the Rhineland in Germany before the COVID pandemic (1 January 2018–30 June 2019) and during the COVID pandemic (1 January 2020–30 June 2021). Shown are the ICD-10 codes and numbers of admitted cases for the 10 most frequent admission diagnoses of the pre-pandemic time period (World Health Organization International Classification of Disorders, 10th revision (https://icd.who.int/browse10/2016/en#/V; last accessed 2 November 2021). Mental disorders with high rates of relative decreases are labeled in brown and one disorder is labeled in red as it showed a relative increase. F10.3, mental and behavioral disorders due to use of alcohol, withdrawal state; F20.0, paranoid schizophrenia; F33.2, recurrent depressive disorder, current episode severe without psychotic symptoms; F32.2, severe depressive episode without psychotic symptoms; F11.2, mental and behavioral disorders due to use of opioids, dependence syndrome; F05.1, delirium superimposed on dementia; F60.31, emotionally unstable personality disorder (borderline type according to the German modification of the ICD-10; https://www.dimdi.de/static/de/klassifikationen/icd/icd-10-gm/kode-suche/htmlgm2021/block-f60-f69.htm); F33.1, recurrent depressive disorder, current episode moderate; F10.3, mental and behavioral disorders due to use of alcohol, withdrawal state; F43.2, adjustment disorders.

We observed increased rates of involuntary admission and involuntary inpatient treatment accompanied by increased rates of coercive measures (restraint and seclusion) in 2020 compared to 2019 (Table 2). Rates were significantly different between the two observation periods for all three variables (two-sided Chi-square-test for all three comparisons *p* < 0.0001 and *df* = 1; PsychKG Chi-square = 280.3; restraint Chi-square = 172.8; seclusion Chi-square = 21.93).

**TABLE 2:** Rates of involuntary psychiatric hospitalization under the auspices of the Mental Health Act (Psych KG NRW) and rates of coercive measures in the nine LVR psychiatric hospitals.

	2018/2019	2020/2021
PsychKG cases	14.3% (*n* = 11,393)	17.5% (*n* = 12,027)
Cases with restraint	3.3% (*n* = 2,622)	3.7% (*n* = 2,573)
Cases with seclusion	3.3% (*n* = 2,631)	4.6% (*n* = 3,186)
Number of admitted patients	79,756	68,894

Numbers in brackets are the numbers of cases of involuntary psychiatric hospitalization under the auspices of the Mental Health Act (Psych KG NRW) and numbers of cases with coercive measures (restraint and seclusion) followed by total numbers of cases in the respective observation periods. Data are presented for the periods January 2018 to June 2019 (before the COVID-19 pandemic) and January 2020 to June 2021 (during the COVID-19 pandemic).

**FIGURE 2:**
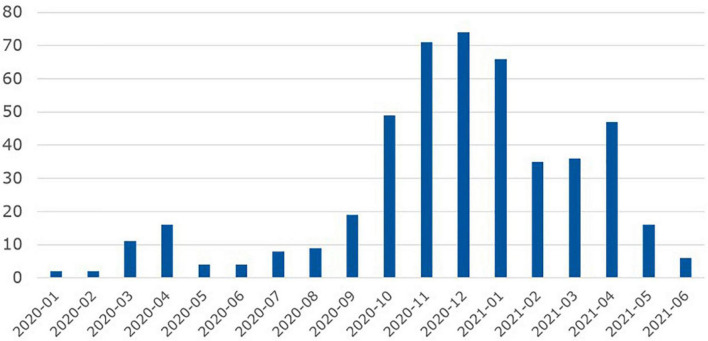
Monthly case numbers of COVID-19 among psychiatric inpatients of the nine psychiatric LVR-hospitals. Observation period: 1 January 2020 until 30 June 2021.

**FIGURE 3:**
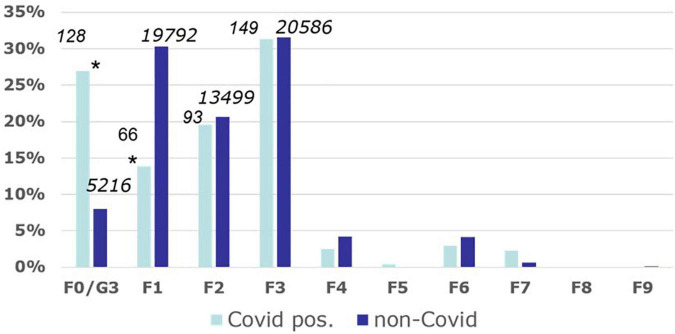
Spectrum of mental disorders of COVID-19-infected psychiatric inpatients. Frequency of COVID-19-cases in different diagnostic groups of psychiatric inpatients of the LVR psychiatric hospitals as differentiated by the World Health Organization International Classification of Disorders, 10th revision (https://icd.who.int/browse10/2016/en#/V; last accessed 2 November 2021). The figures above each bar indicate absolute case numbers during the observation period 1 January 2020 until 30 June 2021. Asterisks indicate that the group differences were significant in Chi square tests (*p* < 0.001). For example: The bars for the diagnostic group F0/G3 (organic mental disorders including dementias) indicate that 27% of all COVID-19-cases, but only 9% of the non-COVID-19-cases, were diagnosed with an F0/G3 disorder. F1, mental and behavioral disorders due to psychoactive substance use; F2, schizophrenia, schizotypal and delusional disorders; F3, mood (affective) disorders; F4, neurotic, stress-related and somatoform disorders; F5, behavioral syndromes associated with physiological disturbances and physical factors; F6, disorders of adult personality and behavior; F7, mental retardation; F8, disorders of psychological development; F9, behavioral and emotional disorders with onset usually occurring in childhood and adolescence.

During the reporting period January 2020 until June 2021, 475 cases of COVID-19 were documented among inpatients of the nine psychiatric hospitals. These cases occurred mainly in the fall and winter months of 2020 and early in 2021 (Figure 2). All COVID-19 cases occurred in inpatients, no cases were observed in day patients. In total, 145 cases were identified between 0 and 4 days after inpatient admission, 97 cases were identified within 5–14 days after admission, and 233 cases were identified later during the course of psychiatric inpatient treatment. Allowing for an incubation period of several days, these figures indicate that the majority of COVID-19 infections (330/475 = 70%) occurred during the inpatient stay. Most psychiatric COVID-19 patients (*n* = 128) were diagnosed with an organic mental disorder including Alzheimer’s dementia (Figure 3). For affective disorders and disorders of the group of schizophrenias, we found similar rates of both infected and non-infected inpatient psychiatric cases. In the group of addiction disorders, COVID-19 cases were relatively underrepresented. The odds ratios for finding a COVID-19 patient in the diagnostic group of the organic mental disorders including the dementias was as high as 4.25 [95%CI 3.44; 5.24], while in the group of addiction disorders, it was only 0.37 [95% CI 0.28; 0.49].

**FIGURE 4:**
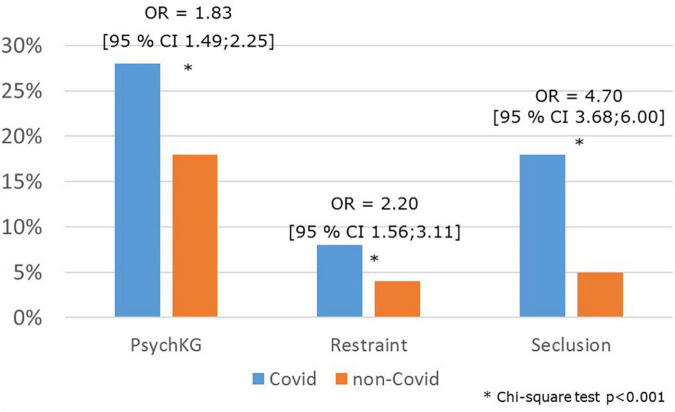
Rates of coercion among psychiatric inpatients with COVID-19. Given are the rates of cases of involuntary hospitalization [Psych KG, indicating the North-Rhine Westphalia Mental Health Act (Psychisch Kranke Gesetz NRW)], and rates of cases with seclusion and restraint in psychiatric inpatients with COVID-19 infections compared to patients without COVID-19 infection. Observation period: 1 January 2020 until 30 June 2021.

Psychiatric inpatients with COVID-19-infections were significantly older (median 58 years, mean ± SD 57 ± 21 years) compared to non-COVID-19 patients (median 46 years, mean ± SD 47 ± 18 years; *t*-test double-sided *p* < 0.0001, T = 10.1507; *df* = 479.02). The relative risk of COVID-19 infection of psychiatric inpatients was significantly increased in women compared to men (relative risk for women 1.32 95% confidence interval 1.100–1.587; Chi-square test *p* < 0.00225; *df* = 1; Chi-Square 9.3374). We found significantly higher rates of involuntary admissions and coercive measures among psychiatric inpatient cases with COVID-19 as compared to non-COVID-19 patient cases (Figure 4).

## Discussion

In this follow up study, we confirm our findings obtained during the first wave of COVID-19 infections in Germany in spring 2020, in which we had shown a reduction of case numbers of psychiatric inpatients (8), which mostly in patients with affective and addiction disorders. We confirm the previously observed trend toward more acute cases, as indicated by diagnostic shifts toward more acute mental illnesses like Acute polymorphic psychotic disorder with symptoms of schizophrenia (ICD-10 F23.1) and increased rates of coercive measures. We further extend our initial findings by showing that these changes were over during beyond the second wave of COVID-19 in late 2020 and early 2021. In addition, we show that the admission rates did not recover during the periods of decreased COVID-19 cases in the summers of 2020 and 2021. This may be due to over during pandemic effects on outpatient consultation rates, which may have led to under assignment of patients to psychiatric inpatient care. Similar findings of an overall decrease of total numbers of unplanned psychiatric hospital admissions were reported from another large German hospital network in 2020 (15, 16). Of note, the diagnostic spectrum was somehow different in that Fasshauer et al. reported that the decrease of unplanned admissions was less pronounced for cases with substance use disorders. This may have been due to a focus on emergency admissions in the studies by Fasshauer et al. compared to all types of hospital admissions in our study.

In addition, we extend our previous findings by showing increased rates of involuntary admissions and coercive measures, which may be due to a relative increase of more severe psychiatric cases during the pandemic and/or the impact of the need to enforce hygiene measures in acute psychiatric wards. In line with our findings, Fasshauer et al. (16) also reported increased rates of involuntary psychiatric admissions and coercive measures in their sample from a large German wide hospital network. Flammer et al. (17), who used the case registry for coercive measures of the state of Baden-Wuerttemberg. Germany, reported similar trends. In contrast, one international study reported declining rates of seclusion and restraint in a Canadian psychiatric hospital during the COVID-19-pandemic (18). Further detailed studies focusing on patient characteristics associated with coercive measures are warranted to identify explanations for such divergent findings.

Especially older people with dementia are a particularly vulnerable group for contracting COVID-19 and the pandemic led to increased mental strains and depression also in caretakers, as was shown in a recent German questionnaire survey of caretakers (19). As in our previous study, there was an overrepresentation of women, people of higher ages and patients with organic mental disorders including dementias among the psychiatric inpatient cases with COVID-19. This finding is now on a firmer empirical basis given the longer observation period and the high number of cases in our present study.

The strengths of our analysis lie in the large numbers of observed cases, the long observation period covering several phases of the pandemic, the new analysis of psychiatric COVID-19 cases and the representativeness of the studied psychiatric hospitals for general psychiatry in Germany. Limitations are the use of routine data documented for clinical and remuneration purposes and the retrospective nature of the study.

Taken together, our study and results from other studies indicate that while psychiatric inpatient case numbers decreased, case severity and disease acuity increased in psychiatric outpatient emergency units (20, 21) and inpatient mental healthcare during the COVID-19 pandemic in Germany. Similar trends were reported from Denmark (9), France (22), Italy (23, 24), Portugal (25), Switzerland (13, 14), and the United Kingdom (26–29) suggesting that these trends are not specific to national mental healthcare services, but reflect more general effects of the pandemic on mental healthcare in Europe. This may have been due to a reduction of mild cases referred to psychiatric services during the pandemic or a reduced rate of use of outpatient services, but it may also indicate that referrals to psychiatric hospitals were deferred until a higher than usual case severity was reached, or that the COVID-19 pandemic led to an increased rate of acute and severe mental disorders in the general population.

An Italian study during the initial COVID-19 pandemic phase showed that the utilization of mental healthcare services increased and was modulated by individual psychosocial factors like the type of coping strategy, indicating that such factors need to be taken into account when preparing mental healthcare services for future pandemics (30). Further large scale follow-up studies in the general population are needed to address these issues. Hence, in response to the experiences during the COVID-19 pandemic the role of psychiatrists and psychiatry are changing with increased responsibilities for ascertaining adequate mental and somatic healthcare for vulnerable people with mental disorders (31, 32).

## Data availability statement

The data analyzed in this study is subject to the following licenses/restrictions: The study was based on anonymized hospital records which cannot be transferred to others due to data protection regulations. Requests to access aggregated statistical analyses should be directed to JZ, juergen.zielasek@lvr.de.

## Ethics statement

Ethical review and approval was not required for the study on human participants in accordance with the local legislation and institutional requirements. Written informed consent for participation was not required for this study in accordance with the national legislation and the institutional requirements.

## Author contributions

JZ and EG-M designed the study. JZ and JV analyzed the data. All authors interpreted the results and wrote the manuscript.
